# Co-occurring Early Adolescent ACEs and Associations With Later Peer Relationships

**DOI:** 10.1007/s10964-025-02157-0

**Published:** 2025-02-13

**Authors:** Joy Huanhuan Wang, Gabriel J. Merrin, Xiafei Wang, Qingyang Liu, Sarah M. Kiefer

**Affiliations:** 1 College of Education, Texas Tech University, Lubbock, TX USA; 2 Department of Human Development and Family Science, Syracuse University, Syracuse, NY USA; 3 School of Social Work, Syracuse University, Syracuse, NY USA; 4 College of Education, University of South Florida, Tampa, FL USA

**Keywords:** Adolescent, Adverse childhood experiences, Peer relationships, Latent class analysis, Trauma-informed care

## Abstract

Research indicates complex associations between adverse childhood experiences (ACEs) and adolescent peer relationships. ACEs are related to lower peer status, yet the links between ACEs, peer characteristics, and peer relationship quality are inconclusive. The current literature has several further conceptual and methodological limitations, including a lack of attention to ACEs’ co-occurring nature, the developmental timing of ACEs during adolescence, and the multifaceted nature of peer relationships. In addition, much of the literature is cross-sectional. The current study addresses these limitations by examining the associations of early adolescents’ co-occurring ACEs at age 12 with three subsequent peer relationship aspects at age 16 (i.e., peer characteristics, peer status, and peer relationship quality) while controlling for demographics and early adversities. Participants included 883 youth from the Longitudinal Studies of Child Abuse and Neglect. Latent class analyses of the 10 ACEs, as examined in the original CDC-Kaiser ACE study, identified three distinct ACE classes at age 12 (threat, deprivation, and low ACEs) that were distinguished by gender, race, income, and early adversities. Further, the threat class was related to adverse outcomes in peer characteristics and status, while the deprivation class was associated with differences in peer relationship quality. These findings highlight the need for researchers and practitioners to consider ACEs’ co-occurring nature and tailor trauma-informed care accordingly. Findings also underscore the salience of studying ACEs that occur in the developmental period of early adolescence.

## Introduction

Research shows adverse childhood experiences (ACEs) have implications for various aspects of adolescent peer relationships, including peer characteristics, peer status, and quality of peer relationships (Henry et al., 2018; Wang et al., 2024a). However, the associations between ACEs and peer relationships are complex; several limitations of the current literature relate to how ACEs are measured, the timing of ACEs studied, and the types of peer relationship outcomes examined. ACEs are often analyzed using either a specificity model, where each ACE is examined separately, or a cumulative model, where ACEs are summed to create a total score. Both approaches fail to capture the co-occurring patterns of ACEs (McLaughlin et al., 2021). Further, most ACE research focuses on the entire childhood (i.e., 0–18 years; lifetime approach) or early childhood (i.e., 0–5 years), with little attention to ACEs in adolescence (Hawes & Allen, 2023). Additionally, few studies have examined the extent to which ACEs are associated with multiple aspects of peer relationships, particularly positive indicators of peer relationships (Wang et al., 2024a). It is critical to understand the associations between ACEs and peer relationships because peers form an essential “community” for the development of youth with ACEs (Substance Abuse and Mental Health Services Administration [SAMHSA], 2014). The current study addresses these gaps by leveraging a person-centered approach to examine co-occurring ACE patterns in early adolescence and their associations with 10 peer relationship outcomes in middle adolescence within a sample of youth experiencing adversity.

### Adverse Childhood Experiences

ACEs are potentially traumatic events that individuals experience throughout childhood (i.e., 0–18 years) and include neglect, abuse, and witnessing domestic violence, among others. The original CDC-Kaiser ACE study examined 10 ACEs (Felitti et al., 1998), although additional ACEs have been considered more recently. ACEs have an enduring impact on human development across life domains, including physical health (e.g., cancer), mental health (e.g., depression), life opportunities (e.g., education), and interpersonal relationships (Hughes et al., 2017; Metzler et al., 2017). ACEs are also common; two-thirds of the U.S. population reported at least one ACE. Further, ACEs often co-occur; 1 in 6 individuals in the U.S. reported experiencing four or more ACEs by age 18 (Swedo et al., 2023).

Traditionally, ACEs have been examined using either the specificity or cumulative models. The specificity model focuses on individual ACEs separately (e.g., physical neglect or sexual abuse) and assumes each ACE has a unique mechanism affecting human well-being. In contrast, the cumulative model assumes a global mechanism across all ACEs and creates a sum score of ACEs to assess dose-response relationships (McLaughlin et al., 2021). Both models have been criticized for notable shortcomings. The specificity model fails to consider that individuals often experience multiple ACEs, which could result in attributing harm to a single ACE when it is a combination of ACEs. The cumulative model assumes that ACEs have additive effects and considers all ACEs as equally influential. In summary, neither approach distinguishes between distinct co-occurring patterns of ACEs and their implications for developmental outcomes (McLaughlin et al., 2021).

Given these shortcomings, viable alternative models have been proposed (Baldwin et al., 2023). For example, the dimensional models offer theoretically driven approaches to distinguish between what is referred to as the “core underlying dimensions” of ACEs (McLaughlin et al., 2021, p. 1464), including threat and deprivation, as outlined in the Dimensional Model of Adversity and Psychopathology (DMAP; McLaughlin et al., 2014). Such dimensions are thought to be generalizable across populations and extend across individual forms of adversity, allowing ACEs to be grouped by the underlying mechanisms by which they affect human development. For example, threat primarily affects emotional processing, whereas deprivation impacts the cognitive system (McLaughlin et al., 2021). The DMAP is supported by the neuroscience literature (McLaughlin et al., 2019). However, evidence supporting its applicability in behavioral science is in its early stages (McLaughlin, 2016).

Another alternative is person-centered methods, such as latent class analysis (LCA; Baldwin et al., 2023), which examine co-occurring ACEs and identify distinct ACE patterns. Studies using LCA suggest ACEs often cluster into 3–5 classes (Wang et al., 2024b). However, the number and nature of the ACE classes, as well as their implications for developmental outcomes, are often specific to the study samples and the ACE indicators used (Baldwin et al., 2023). Such variability highlights the need for additional LCA research that analyzes established ACE indicators and uses theoretical frameworks when naming ACE classes to enhance comparability across studies and facilitate a clearer interpretation of observed variability. It also underscores the need to examine diverse and specialized samples (e.g., at-risk vs. community samples) to expand knowledge on how ACEs cluster together across different groups. Therefore, this study analyzed a diverse at-risk sample, utilized the 10 ACEs as defined in the CDC-Kaiser ACE study to model the ACE classes, and referenced the DMAP in naming and interpreting the ACE classes.

ACE research has traditionally assessed adversities throughout childhood (i.e., 0–18 years) or focused on early childhood (i.e., 0–5 years; Felitti et al., 1998; Hawes & Allen, 2023). ACEs in adolescence are understudied (Hawes & Allen, 2023). However, recent research underscores the importance of adopting a developmental life course perspective by examining ACEs occurring in later developmental periods, such as adolescence (Logan-Greene et al., 2024). Developmental timing plays a critical role in understanding both the mechanisms through which ACEs affect various biopsychosocial outcomes and the extent to which the co-occurrence of ACEs may vary across developmental stages (Hawes & Allen, 2023; Reh et al., 2020). Early adolescence is a critical developmental period (Dahl et al., 2018) characterized by unique features like neuroplasticity in puberty, which can influence how ACEs affect developmental outcomes. For example, this stage presents an opportunity to recalibrate the hypothalamic-pituitary-adrenal axis, which may have been affected by early adversities (DePasquale et al., 2021). Further, preliminary empirical research indicates ACEs experienced during early adolescence can have distinct developmental implications than those experienced during early childhood (Logan-Greene et al., 2024). Notably, ACEs in later developmental periods are related to early adversities (Brown et al., 2019). Thus, the current study examined ACEs experienced during early adolescence (i.e., around age 12) while controlling for early adversities.

Further, ACEs’ prevalence rates and implications for developmental outcomes vary across demographic variables, including gender, race, and family income (Beyer et al., 2015; Felitti et al., 1998; Yang et al., 2025). Women, racial and ethnic minorities, and individuals with low family income are more likely to experience high rates of ACEs (Felitti et al., 1998; Swedo et al., 2023). However, the associations between ACE classes and demographic variables are less clear, partially due to the variability of ACE classes across studies (Wang et al., 2024b). Nonetheless, recent research provided preliminary evidence on the associations between demographic variables and ACE classes. For instance, women tend to have more complex ACE profiles than men (Haahr-Pedersen et al., 2020), Black and Latinx youth tend to have different ACE profiles than White youth (Maguire-Jack et al., 2020), and low family income is related to high-ACE profiles (Liu et al., 2018). To add to the emerging literature on demographics and ACE classes, this study investigated age, gender, race, income, and geographic locations as demographic characteristics of ACE classes and further controlled them in examining the associations between ACE classes and peer relationships.

### Associations Between ACEs and Adolescent Peer Relationships

Peer relationships are critical for adolescent well-being across life domains, both in the general population and particularly among youth with ACEs (Bukowski et al., 2018; Wang et al., 2024a). Peers often serve as a first line of support, providing a safe space for youth to disclose ACEs, such as sexual abuse, physical abuse, and neglect (Wang et al., 2024a). Further, peers form an essential “community” that facilitates trauma recovery for youth (SAMHSA, 2014). However, youth with ACEs are more likely to experience negative peer relationships, such as fewer friends and lower peer status (Wang et al., 2024a). According to the social learning theory (Bandura, 1977), home environments are a critical socializing agent that guides youth’s social attitudes and behaviors. When experiencing adversities in their interactions with families, youth may observe inappropriate behaviors (e.g., aggression) as well as experience emotional dysregulation and lack of trust, which can challenge them from developing healthy interpersonal relationships, including peer relationships (McLaughlin et al., 2021; Poole et al., 2018). Nonetheless, research suggests ACEs may have differential implications for various aspects of peer relationships. For instance, ACEs are related to lower peer status, yet the associations between ACEs, peer characteristics, and peer relationship quality are less conclusive (Wang et al., 2024a).

Further, the literature has several notable gaps that warrant investigation (Wang et al., 2024a). Similar to the broader ACE literature, research on ACEs and peer relationships often examines individual ACEs in isolation (i.e., the specificity model). This area of research lags even further behind the broader ACE literature, as the cumulative model is rarely used, and person-centered approaches have yet to be widely used. This notable limitation prevents a comprehensive understanding of how distinct co-occurring ACE patterns are associated with adolescent peer relationships. Further, much of the literature on ACEs and peer relationships is cross-sectional, which obscures understanding of how ACEs are associated with peer relationships over time. There is also a need to examine multiple aspects of peer relationships with ACEs rather than focusing on a single peer relationship outcome. To address these gaps, the current study used a person-centered approach to examine ACE classes at age 12 and relate them to three salient aspects of peer relationships at age 16 (i.e., peer characteristics, peer relationship quality, and peer status).

Peer characteristics refer to the behaviors, features, and traits of the peers with whom youth are affiliated, including positive (e.g., prosocial peers) and negative (e.g., peer delinquency) characteristics (Wang et al., 2024a). These characteristics have implications for the well-being of youth with ACEs. For example, positive peer characteristics may help mitigate the adverse effects of ACEs on youth development (Garrido & Taussig, 2013), whereas negative peer characteristics can exacerbate ACEs’ impact (Henry et al., 2018). In addition, peer characteristics may serve as a mechanism (i.e., mediation) by which ACEs further influence youth outcomes, such as substance use (Feske et al., 2008). Nonetheless, the literature is inconclusive regarding the associations between ACEs and peer characteristics. For instance, Wang et al. (2024a) found in a systematic review that 17 studies reported ACEs were related to negative peer characteristics, such as deviant peer affiliation and peer violence and substance use. Meanwhile, 12 studies suggested nonsignificant associations, and one study found that ACEs were related to positive peer characteristics (i.e., less peer alcohol use). Notably, much of this literature examined ACEs through the specificity model without considering ACEs’ co-occurring nature. In addition, research focuses on the links between ACEs and negative peer characteristics, with limited attention to positive ones. Adolescents often engage with both positive and negative peers, who may affect their development through distinct mechanisms (e.g., Walters, 2020). Therefore, this study examined positive and negative peer characteristics (i.e., positive peer behavior, peer substance use, and peer delinquency) in relation to the co-occurring ACE patterns.

Peer relationship quality refers to the quality of interactions among similar-aged youth and also has a critical impact on the development of youth with ACEs, such as mental health and delinquency (Ban & Oh, 2016). Similar to peer characteristics, the literature on ACEs and peer relationship quality is inconclusive. In the same review, Wang et al. (2024a) found that 16 studies reported ACEs were negatively related to peer relationship quality, whereas 13 studies suggested nonsignificant associations and one indicated a positive association. Further, much of the literature conceptualizes ACEs via the specificity model and often examines the presence of positive indicators (e.g., intimacy) or the absence of negative features (e.g., conflict) of peer relation quality in the context of ACEs. In reality, these two types of indicators can co-occur (e.g., a close relationship can have high levels of intimacy and conflict; Laurenceau et al., 2005), and both can affect youth development. Thus, the current study analyzed both positive and negative indicators of peer relationship quality (i.e., companionship, satisfaction, intimacy, and conflict) in relation to the co-occurring ACE patterns.

Peer status refers to the social position youth hold among peers and is another potential mechanism through which ACEs affect youth development. For example, ACEs may be associated with lower peer status, which in turn negatively affects various youth outcomes, such as depression (Alto et al., 2018) and violence (Chapple et al., 2005). Further, lower peer status might exacerbate ACEs’ negative influences on youth development (Christ et al., 2017). In contrast to peer characteristics and peer relationship quality, the literature is conclusive that ACEs are related to lower peer status. However, this conclusion is based on studies that primarily conceptualized and measured ACEs through the specificity model without capturing ACEs’ co-occurring nature (Wang et al., 2024a). Thus, the current study examined the implications of co-occurring ACE patterns for peer status (i.e., peer popularity, aggression, and victimization) to gain a more holistic understanding.

## Current Study

**Fig. 1:**
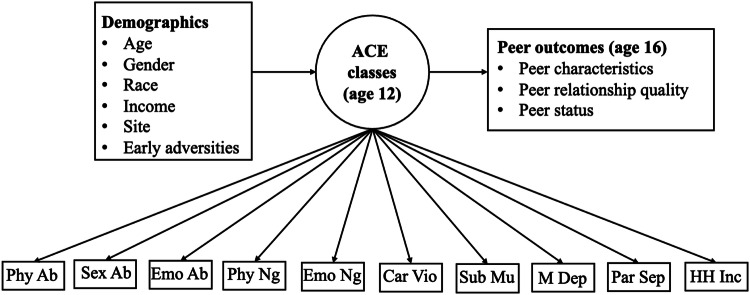
Hypothesized multivariate model. ACE adverse childhood experiences, Phy Ab physical abuse, Sex Ab sexual abuse, Emo Ab emotional abuse, Phy Ng physical neglect, Emo Ng emotional neglect, Car Vio caregiver being treated violently, Sub Mu family substance misuse, M Dep maternal depressive symptoms, Par Sep parental separation, HH Inc household incarceration

Research suggests ACEs are related to lower adolescent peer status, yet the evidence is less conclusive regarding the associations between ACEs, peer characteristics, and peer relationship quality. Further, several gaps persist in the literature: (1) lacking consideration of ACEs’ co-occurring nature, (2) limited research on ACEs that occur in adolescence, (3) lacking longitudinal studies, and (4) limited attention to the multifaceted nature of peer relationships, particularly positive indicators. To address these gaps, the current study conducted latent class analysis in a sample of at-risk youth to identify ACE classes at age 12 and explore their implications for peer relationships at age 16 (see Fig. 1). Specifically, this study addressed three questions. What ACE classes emerge at age 12 (Research Question 1)? Which demographic variables characterize these classes (Research Question 2)? How are the ACE classes related to peer relationships at age 16, with early adversities and demographic variables being controlled (Research Question 3)? The research questions were exploratory, considering the understudied nature of early adolescent ACE classes and their implications for later peer relationships.

## Methods

### Participants and Procedures

Participants in the current study were from the Longitudinal Studies of Child Abuse and Neglect (LONGSCAN), a multi-site study that examines the effects of maltreatment on child development (Runyan et al., 2014). Participants were recruited from five geographic sites across the U.S. (i.e., East, Midwest, Northwest, South, and Southwest) and were identified as at-risk for or having a history of child maltreatment. Data were collected through surveys and administrative records from the youth, teachers, and home caregivers. The baseline sample included 1354 youth, who were followed longitudinally from early childhood through adolescence, with data collection waves at roughly ages 4, 6, 8, 12, 14, 16, and 18. For the current study, the analytic sample consisted of 883 youth with available data at ages 12 and 16. The demographic variables of the analytic sample are very similar to those of the baseline sample (see Supplemental Table S1). The reduced sample size, compared to age 4, was primarily due to follow-up losses, as the sample was recruited from a highly mobile population (Du & Kim, 2021).

### Measures

Aligned with the CDC-Kaiser ACE study, 10 ACEs were identified from the LONSCAN dataset. Further, three aspects of peer relationships were measured—peer characteristics, peer relationship quality, and peer status—each with multiple indicators. Higher scores indicate higher levels of each ACE or the specific peer relationship variable (see Table 1 and Supplemental Appendix A for more measure information). ACEs assessed at age 12 were selected because this is the only time point in *early adolescence* with available data on all 10 ACEs. Age 16 was chosen for peer relationship variables because this is the only time point after age 12 that has data on all peer relationship variables of interest, which allowed for examining the associations between ACE classes and peer relationships across two waves.

**Table 1:** ACE and peer relationship measures

Variable	Instrument	Time frame	Respondent	Item No.	Site
Physical abuse	PHYA^a^	Last year	Youth	16	All
Sexual abuse	SARA^a^	Last year	Youth	12	All
Emotional abuse	PSMA^a^	Last year	Youth	20	All
Physical neglect	About My Parents^b^	Last year	Youth	7	All
Emotional neglect	About My Parents^b^	Last year	Youth	7	All
Caregiver treated violently	CTS2^c^	Last year	Caregiver	12	All
Family substance misuse	RBFA^a^	Current	Youth	5	All
Maternal depressive symptoms	CES-D^d^	Past week	Caregiver	20	All
Parental separation	Family Chart^a^	Last year	Caregiver	2	All
Household incarceration	Child Life Events^a^	Last year	Caregiver	1	All
Peer substance use	RBFA^a^	Current	Youth	6	All
Peer delinquency	RBFA^a^	Current	Youth	7	All
Positive peer behavior	RBFA^a^	Current	Youth	5	All
Peer popularity	TEPS^e^	Current	Teacher	4	EA, NW, SW
Peer aggression	TEPS^e^	Current	Teacher	2	EA, NW, SW
Peer victimization	YPV^f^	Since age 12	Youth	11	All
Peer companionship	NRI^g^	Current	Youth	3	All
Peer conflict	NRI^g^	Current	Youth	3	All
Peer satisfaction	NRI^g^	Current	Youth	3	All
Peer intimacy	NRI^g^	Current	Youth	3	All

*ACE* adverse childhood experiences, *CES-D* center for epidemiologic studies depression, *CTS*2 the revised conflict tactics scales, *EA* east, *No*. number, *NRI* network of relationships inventory, *NW* northwest, *PHYA* self-report of physical abuse and assault, *PSMA* self-report of psychological maltreatment, *RBFA* risk behaviors of family and friends, *SARA* self-report of sexual abuse and assault, *SW* southwest, *TEPS* teacher’s estimation of peer status, *YPV* youth peer victimization

^a^Developed by LONSCAN

^b^Modified from The Neglect Scale (Straus et al., 1997)

^c^Straus et al. (1996)

^d^Radloff (1977)

^e^Lemerise & Dodge (1990)

^f^Modified from the Juvenile Victimization Questionnaire (Finkelhor et al., 2005)

^g^Modified from Furman and Buhrmester (1985)

#### Physical, sexual, and emotional abuse

The Self-Report of Abuse and Assault (physical, sexual, and psychological) developed by LONGSCAN was used for youth to report experiences of abuse in these three areas in the past year. Physical and sexual abuse were rated on a 4-point scale, from 0 (*never*), 1 (*1 time*), 2 (*2 or 3 times*), to 3 (≥*4 times*). Emotional abuse was assessed on a 3-point scale, from 0 (*never*), 1 (*sometimes*), to 2 (*often*). Item responses can be either summed for each scale or dichotomized and summed. This study dichotomized item responses (i.e., 0 as 0; 1–3 as 1 for physical and sexual abuse; 1–2 as 1 for emotional abuse) before summing to create a total score for each type of abuse. The physical abuse scale included 16 items, such as adults hitting youth with a baseball bat. The sexual abuse scale contained 12 items, such as people putting their body parts inside the youth’s bottom. Lastly, the emotional abuse scale was comprised of 20 items, such as parents making youth feel bad about themselves.

#### Physical and emotional neglect

Youth reported their experiences with physical needs and emotional support in the past year using the About My Parents scale, modified by LONGSCAN from The Neglect Scale (Straus et al., 1997). Items were rated on a 4-point scale, from 0 (*never*), 1 (*almost never*), 2 (*sometimes*), to 3 (*a lot*). The physical needs subscale included seven items, with examples like parents giving youth enough clothes to keep warm or making sure youth bathe regularly. The emotional support subscale also contained seven items, such as parents doing things with youth just for fun. Since all items were positively worded, they were reverse-coded to reflect parents’ neglectful behaviors. Item responses were then dichotomized (i.e., 0–1 as 0; 2–3 as 1) and summed to create a total score for physical and emotional neglect, respectively. Notably, only the top half of the reverse-coded item responses (i.e., scores of 2–3) were coded as 1 because the scale includes scenarios that may not be deemed high risk by the Child Protective Services (e.g., Kobulsky et al., 2018).

#### Caregiver being treated violently

Maternal primary caregivers reported partner-to-respondent violence in the past year using the physical assault subscale of the Revised Conflict Tactics Scales: Partner-to-Partner (Straus et al., 1996). The subscale contained 12 items, such as “My partner twisted my arm” or “My partner used a knife or gun on me.” Maternal caregivers rated the frequency of such behaviors on a 4-point scale, from 0 (*never*), 1 (*1 time*), 2 (*2–5 times*), to 3 (>*5 times*). Responses were dichotomized (i.e., 0 as 0; 1–3 as 1) and then summed to create a total score.

#### Family substance misuse

Youth reported the current substance misuse of their families via five items of the Risk Behaviors of Family and Friends scale, which was developed by LONGSCAN. All items started with the stem of “Does anyone that you live with …” and they assessed the following substances: marijuana, cocaine, meth, injected drugs, and getting drunk. Items were rated as 0 (*no*) or 1 (*yes*) and then summed to create a total score of family substance misuse.

#### Maternal depressive symptoms

Maternal primary caregivers reported their depressive symptoms in the past week using the Center for Epidemiologic Studies Depression Scale (CES-D; Radloff, 1977). The scale included 20 items (e.g., “I had crying spells”) that were rated on a 4-point scale, from 0 (*rarely or none of the time*), 1 (*a little of the time*), 2 (*a moderate amount of the time*), to 3 (*most or all of the time*). Four items were positively worded (e.g., “I felt hopeful about the future”) and reverse-coded before summing. The total score has a possible range of 0–60. It was further dichotomized using 16 as a cut-off point (i.e., 0–15 as 0; ≥16 as 1) for clinically elevated depressive symptoms following the literature on CES-D (Radloff, 1977).

#### Parental separation

Primary maternal caregivers reported their household composition through the Family Chart developed by LONSCAN, which collected information on up to 14 persons who lived in the same house with the youth participant and their relationships with the youth (see Supplemental Appendix A). The youth’s family structure (i.e., whether living with two biological parents or not) was used as a proxy for parental separation.

#### Household incarceration

Primary caregivers reported the household incarceration in the past year for the youth’s father (or father figure), mother (or mother figure), or another family member in the household using the Child’s Life Events scale developed by LONGSCAN. The item asked, “Who was jailed, imprisoned, or kept in a juvenile residential facility for breaking the law?” Items were rated as 0 (*no*) or 1 (*yes*) and were summed across the three family members, with a possible range of 0–3, which was further dichotomized to create a binary indicator of household incarceration (0 for no incarceration; 1 for any incarceration).

#### Peer characteristics

Youth reported three peer characteristics using the Risk Behaviors of Family and Friends scale developed by LONSCAN, including peer substance use, peer delinquency, and positive peer behaviors. All questions started with the stem of “How many of your close friends …” and were rated via a 3-point scale, including 0 (*none of my friends*), 1 (*some of my friends*), and 2 (*most of my friends*). Responses were dichotomized (i.e., 0 as 0; 1–2 as 1) and then summed for each subscale. The peer substance use subscale included six items, assessing youth perceptions of their friends’ use of the following substances: cigarettes, alcohol, marijuana, cocaine, heroin, and other drugs. The peer delinquency subscale was comprised of seven questions, measuring youth perceptions of their friends’ engagement in the following risky behaviors: sexual intercourse, carrying weapons, selling or delivering drugs, stealing, setting fires, fighting, and damaging properties. Lastly, the positive peer behavior subscale contained five items, assessing youth perceptions of their friends’ prosocial behaviors (i.e., participation in church, school clubs, and sports) and school performance (i.e., earning good grades and behaving well).

#### Peer relationship quality

Youth reported four indicators of peer relationship quality using the Network of Relationships Inventory, adapted from Furman and Buhrmester (1985), including companionship (e.g., having fun with friends), conflict (e.g., getting upset with friends), satisfaction (e.g., happy with the friendship), and intimacy (e.g., telling friends everything). Each indicator had three items, and all items were rated on a 5-point scale. Further, each item branched for six follow-up items, two of which assessed the relationship quality with (1) “best male friend who is not a brother or boyfriend” and (2) “best female friend who is not a sister or girlfriend.” The higher value of these two follow-up items was selected to represent each item. Then, an average score was calculated for each indicator.

#### Peer status

Three indicators of peer status were assessed. Teachers rated youth’s peer popularity and aggression using the Teacher’s Estimation of Peer Status (Lemerise & Dodge, 1990). The peer popularity subscale had four items, e.g., would be nominated by classmates for “would like most for play or work partner.” The peer aggression subscale included two items, e.g., would be nominated by classmates as “starts arguments or fights.” Youth reported their experiences of physical peer victimization (11 items, e.g., “cut or scraped you”) using the Youth Peer Victimization scale, which was modified by LONGSCAN from the Juvenile Victimization Questionnaire (Finkelhor et al., 2005). Peer popularity and aggression were rated on a 5-point scale from 0 to 4. Most items were reverse-coded, and average scores for the peer popularity and aggression subscales were calculated. Peer victimization items were rated as 0 (*no*) or 1 (*yes*) and then summed. Notably, data on the victimization caused by “another kid outside of your household or family” and “a group of kids” were extracted for analyses (see Supplemental Appendix A).

#### Control variables

Demographic variables included the child’s age, gender (i.e., male, female), race/ethnicity (i.e., Black, White, Hispanic, multiracial, and other race), income, and geographic sites. Race/ethnicity and geographic sites were dummy-coded. Household income at age 12 ranged from 0 (*less than $5000 per year*) to 10 *($50,000 per year or more*). Further, the youth’s early adversities were controlled, including physical, sexual, and emotional abuse, as well as physical and emotional neglect. They were assessed via the same instruments as those described above for measuring abuse and neglect at age 12, except that data on the epoch before elementary school were selected for early physical, sexual, and emotional abuse, and data on the epoch of elementary school were chosen for early physical and emotional neglect. Responses to each early adversity measure were dichotomized and then summed to create a total score for early adversities.

### Analysis Plan

Latent class analysis (LCA) and multinomial and linear regression were conducted to address the research questions. First, a class enumeration process using the 10 ACEs was conducted to determine the number and nature of the ACE classes. Next, multinomial logistic regression was used to better understand the characteristics of individuals within each ACE class. In the final step, linear regression was conducted to examine the associations between the ACE classes and 10 peer relationship outcomes, while controlling for demographic variables and early adversities. The 1–5 latent class solutions were evaluated using a combination of fit indices and substantive interpretation (Nylund et al., 2007). The fit indices included −2 Log Likelihood (−2LL), Akaike Information Criterion (AIC), Bayesian Information Criterion (BIC), Consistent Akaike Information Criterion (CAIC), Approximate Weight of Evidence Criterion (AWE), the adjusted Lo-Mendell-Rubin likelihood ratio test (LMRT), and entropy (i.e., degree of class separation). The adjusted LMRT compares the current latent class model to a model with one fewer class (e.g., comparing a 3-class to a 2-class model). Entropy values were used to evaluate classification quality, where values closer to one indicate a higher degree of certainty in class membership (i.e., modal class assignment). The manual three-step approach was applied to minimize class shifting when adding predictors, which uses logits to fix individuals in the classes. Further, the BCH method (Asparouhov & Muthén, 2021), which uses weighted multiple-group analysis, was used to prevent the classes from shifting when outcomes were added to the model. All models were examined in Mplus 8.11 using the robust Maximum Likelihood Estimator (MLR).

Regarding missing data, two peer relationship variables (i.e., peer popularity and aggression) were assessed at three of the five sites, and the remaining variables were measured at all sites (see Table 1). While overall attrition from age 12 to 16 was modest (*n* = 883 vs. *n* = 762, approximately 14%), the Missing at Random (MAR) assumption was assessed, namely, whether the missingness on key peer relationship outcomes was associated with demographic variables. Binary indicators (0 = missing, 1 = observed) for each of the 10 peer relationship variables at age 16 were created, and chi-square tests were conducted with gender, race, and site. Results showed no significant differences between missingness on peer outcomes and gender, which means that missingness was not systematically related to gender. However, missingness did differ significantly by race for several outcomes: peer substance use (χ^2^ = 12.53, *df* = 4, *p* = 0.014), peer delinquency (χ^2^ = 12.53, *df* = 4, *p* = 0.014), positive peer behavior (χ^2^ = 12.53, *df* = 4, *p* = 0.014), peer popularity (χ^2^ = 11.19, *df* = 4, *p* = 0.024), peer aggression (χ^2^ = 11.19, *df* = 4, *p* = 0.024), and peer victimization (χ^2^ = 12.91, *df* = 4, *p* = 0.012). Further, missingness differed by site for nearly all peer relationship variables (*p* < 0.001). In summary, the chi-square tests suggested that missingness might be systematically related to race and site. To address any potential bias due to missing data on these variables, race and site were included as control variables in all models. As such, the models accounted for any demographic-driven patterns of missingness and helped ensure that the parameter estimates remained robust under the MAR assumption. Further, missing data were handled with Full Information Maximum Likelihood (vs. listwise deletion), which uses all available data by treating observed indicators as latent factors, allowing each individual to contribute whatever available data to the likelihood function.

## Results

### Descriptives

Descriptive statistics for the analytic sample can be found in Table 2. The study included 883 youth with an average age of 12.37 years at the time of data collection, referred to as “age 12” to reflect the approximate age of participants. The sample was evenly split by gender, with 50.3% identifying as female. Regarding racial composition, 55.2% of the participants identified as Black, 6.3% as Hispanic, 11.3% as Multiracial, 1.2% as Other, and 25.9% as White. The average family income was 4.86 on a scale from 0 to 10, where a score of 4 indicated an annual household income between $15,000 and $19,999, and a score of 5 indicated a yearly income between $20,000 and $24,999.

**Table 2:** Descriptive statistics of study variables (*N* = 883)

Variable	Mean (or *n*) & *SD* (or %)	Range	ACE frequency						
			0	1	2	3	4	5	6
Age	12.37 (0.44)	11.01–14.18							
Gender									
Female	444 (50.3%)								
Male	439 (49.7%)								
Race									
Black	487 (55.2%)								
Hispanic	56 (6.3%)								
Multiracial	100 (11.3%)								
Other	11 (1.2%)								
White	229 (25.9%)								
Income	4.86 (3.13)	0.00–10.00							
Site									
East	185 (21.0%)								
Midwest	132 (14.9%)								
Northwest	176 (19.9%)								
South	164 (18.6%)								
Southwest	226 (25.6%)								
Early adversities			377	252	134	82	26	12	
Physical abuse			593	133	84	69^a^			
Sexual abuse			780	33	16	43^a^			
Emotional abuse			565	87	62	37	30	17	82^a^
Physical neglect			778	42	63^a^				
Emotional neglect			567	84	107	29	96^a^		
Caregiver treated violently			592	40	16	36^a^			
Family substance misuse			754	85	42^a^				
Maternal depressive symptoms			621	220^b^					
Parental separation			117	746^b^					
Household incarceration			766	98^b^					
Peer substance use	1.60 (1.43)	0.00–6.00							
Peer delinquency	2.06 (1.68)	0.00–7.00							
Positive peer behavior	4.46 (0.96)	0.00–5.00							
Peer popularity	2.20 (0.80)	0.00–4.00							
Peer aggression	1.16 (1.21)	0.00–4.00							
Peer victimization	0.36 (0.90)	0.00–7.00							
Peer companionship	1.94 (0.88)	0.00–4.00							
Peer conflict	1.10 (0.81)	0.00–4.00							
Peer satisfaction	2.90 (0.76)	0.00–4.00							
Peer intimacy	1.89 (1.07)	0.00–4.00							

^a^The participants with a higher number of the corresponding ACE are combined into the current group. For example, 69 participants scored three or more on physical abuse

^b^Maternal depressive symptoms, parental separation, and household incarceration were assessed as No or Yes

### What ACE Classes Emerged at Age 12?

**Fig. 2:**
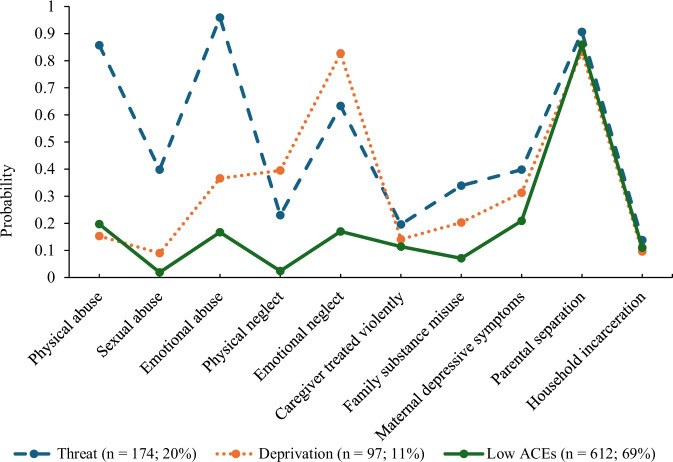
Plotted latent class solution of 10 ACEs. ACE adverse childhood experiences

The class enumeration began with a 1-class solution and sequentially added classes while assessing model fit. This process ended when the adjusted LMRT was no longer significant. Five models were evaluated, with the 3-class solution showing the second-best fit and the best substantive interpretability (see Table 3). This 3-class solution had an entropy value of 0.777, indicating an adequate degree of class separation and certainty in modal class assignments. The 4-class solution, although having a slightly better model fit, included a class with fewer than 40 individuals, making it too small for further analyses. Thus, the 3-class solution was selected as the final model for subsequent analyses. The three classes included *low* (*n* = 612; 69%), *threat* (*n* = 174; 20%), and *deprivation* (*n* = 97; 11%) ACE classes (see Fig. 2). The *low* ACE class was characterized by low endorsement probabilities of all 10 ACEs except parental separation. The *threat* class was distinguished by the highest probabilities of physical, sexual, and emotional abuse, as well as family substance misuse and maternal depressive symptoms. Lastly, the *deprivation* class was characterized by the highest probabilities of physical and emotional neglect. The sample had limited variability on a number of ACEs, including parental separation, household incarceration, and caregiver being treated violently. The endorsement probabilities of each ACE across the three classes are provided in Supplemental Fig. S1.

**Table 3:** Fit statistics for the 1–5 class solutions

Class number	−2LL	AIC	BIC	CAIC	AWE	Adjusted LMRT	Adjusted LMRT *p*-value	Entropy
1-Class	11,324.23	11,376.23	11,500.60	11,526.60	11,754.97	-	-	1
2-Class	10,799.82	10,905.82	11,159.34	11,212.34	11,677.85	521.21	0.001	0.772
**3-Class**	**10,692.01**	**10,852.01**	**11,234.68**	**11,314.68**	**12,017.35**	**107.22**	**0.0589**	**0.777**
4-Class	10,606.15	10,820.15	11,331.96	11,438.96	12,378.78	85.40	0.0509	0.788
5-Class	10,536.22	10,804.22	11,445.19	11,579.19	12,756.15	69.55	0.761	0.811

Bold indicates the final solution adopted

*−2LL* −2 log likelihood, *AIC* akaike information criterion, *AWE* approximate weight of evidence criterion, *BIC* bayesian information criterion, *CAIC* consistent akaike information criterion, *LMRT* lo-mendell-rubin likelihood ratio test

### What Demographic Variables Characterized These ACE Classes?

Multinomial logistic regression was conducted to examine the associations between demographic variables and ACE class membership. Odds ratios (OR) and 95% confidence intervals (CI) for the three class comparisons are presented in Table 4. Compared to males, females had lower odds of being in the threat class (*OR* = 0.43, 95% CI [0.19, 0.98], *p* = 0.045) than the low ACE class. Compared to their White counterparts, Black youth had lower odds of being in the threat (*OR* = 0.35, 95% CI [0.13, 0.91], *p* = 0.031) and deprivation (*OR* = 0.15, 95% CI [0.02, 0.99], *p* = 0.050) classes than the low ACE class. No other significant racial differences were found. Having higher household income was related to lower odds of being in the deprivation class (*OR* = 0.78, 95% CI [0.64, 0.94], *p* = 0.011) than the low ACE class and lower odds of being in the threat class (*OR* = 0.83, 95% CI [0.71, 0.98], *p* = 0.031) than the deprivation class. Experiencing ACEs before age 12 was related to higher odds of being in both the threat (*OR* = 20.02, 95% CI [9.36, 42.82], *p* < 0.001) and deprivation (*OR* = 115.96, 95% CI [27.79, 483.95], *p* < 0.001) classes than the low ACE class and higher odds of being in the threat (*OR* = 5.79, 95% CI [2.10, 16.00], *p* < 0.001) than the deprivation class. Notably, the *ORs* for the early adversity variable are inflated when comparing the threat and deprivation classes to the low ACE class due to the very low levels of ACE exposure in the low ACE class. However, the comparisons between the threat and deprivation classes are not inflated, and the observed differences are meaningful. Neither age nor geographic sites were related to ACE class membership.

**Table 4:** Odds ratios and 95% CI of demographic variables and early adversities on ACE classes

Variable	Threat vs. Low	Deprivation vs. Low	Threat vs. Deprivation
Age	1.07 [0.29, 3.92]	2.57 [0.30, 22.09]	2.41 [0.34, 16.88]
Gender	0.43^*^ [0.19, 0.98]	0.94 [0.25, 3.48]	2.18 [0.81, 5.90]
Black	0.35^*^ [0.13, 0.91]	0.15^*^ [0.02, 0.99]	0.43 [0.10, 1.96]
Hispanic	2.58 [0.65, 10.21]	0.40 [0.01, 11.56]	0.15 [0.01, 3.56]
Multiracial	0.32 [0.06, 1.69]	0.13 [0.01, 1.16]	0.40 [0.07, 2.31]
Other race	1.16 [0.04, 33.58]	0.20 [0.01, 6.05]	0.17 [0.02, 1.34]
Income	0.93 [0.81, 1.06]	0.78^*^ [0.64, 0.94]	0.83^*^ [0.71, 0.98]
Site: East	2.24 [0.47, 10.68]	1.77 [0.17, 18.49]	0.79 [0.11, 5.81]
Site: Northwest	3.26 [0.83, 12.77]	1.83 [0.17, 19.95]	0.56 [0.07, 4.50]
Site: South	3.18 [0.74, 13.72]	3.00 [0.32, 28.43]	0.95 [0.11, 7.99]
Site: Southwest	2.17 [0.48, 9.83]	1.57 [0.16, 15.60]	0.73 [0.09, 5.74]
Early adversities	20.02^***^ [9.36, 42.82]	115.96^***^ [27.79, 483.95]	5.79^***^ [2.10, 16.00]

Male is the reference category for the gender variable. White is the reference category for the race variables. Midwest is the reference category for the site variables. Low represents the Low ACEs class

*CI* confidence intervals

^*^p < 0.05, ^***^p < 0.001

### How Were the ACE Classes Related to Peer Relationships at Age 16?

Linear regression was conducted to examine the associations between the three ACE classes and 10 peer relationship outcomes. The peer relationship variables were organized into three distinct domains and analyzed separately. All models controlled for demographic variables and early adversities. Table 5 presents the means, standard errors, and *p*-values for each peer relationship outcome. The results are also plotted in Supplemental Fig. S2. Marginal effects with *p*-values close to significance (i.e., 0.059 and 0.063) are considered meaningful, given the small sample sizes for the threat (*n* = 174) and deprivation (*n* = 97) classes.

**Table 5:** Means and standard errors of ACE classes at age 12 on peer relationships at age 16

Variable	Threat	Deprivation	Low	Chi-square comparison
Peer characteristics				
Peer substance use	1.89 (0.15)	1.72 (0.21)	1.48 (0.07)	T vs. D, *p* = 0.532 **T vs. L,** ***p*** = **0.011** D vs. L, *p* = 0.272
Peer delinquency	2.53 (0.20)	2.25 (0.24)	1.86 (0.08)	T vs. D, *p* = 0.415 **T vs. L,** ***p*** = **0.002** D vs. L, *p* = 0.146
Positive peer behavior	4.28 (0.11)	4.49 (0.12)	4.51 (0.05)	T vs. D, *p* = 0.239 **T vs. L,** ***p*** = **0.059** D vs. L, *p* = 0.845
Peer status				
Peer popularity	1.97 (0.09)	2.10 (0.14)	2.31 (0.05)	T vs. D, *p* = 0.459 **T vs. L,** ***p*** = **0.001** D vs. L, *p* = 0.168
Peer aggression	1.39 (0.14)	1.18 (0.19)	1.07 (0.07)	T vs. D, *p* = 0.410 **T vs. L,** ***p*** = **0.041** D vs. L, *p* = 0.592
Peer victimization	0.69 (0.12)	0.37 (0.14)	0.24 (0.04)	T vs. D, *p* = 0.106 **T vs. L,** ***p*** = **0.001** D vs. L, *p* = 0.390
Peer relationship quality				
Peer companionship	1.92 (0.09)	1.62 (0.12)	1.99 (0.05)	**T vs. D,** ***p*** = **0.063** T vs. L, *p* = 0.462 **D vs. L,** ***p*** = **0.004**
Peer conflict	1.24 (0.09)	0.85 (0.09)	1.13 (0.04)	**T vs. D,** ***p*** = **0.005** T vs. L, *p* = 0.268 **D vs. L,** ***p*** = **0.009**
Peer satisfaction	2.97 (0.08)	2.63 (0.11)	2.93 (0.04)	**T vs. D,** ***p*** = **0.021** T vs. L, *p* = 0.640 **D vs. L,** ***p*** = **0.015**
Peer intimacy	1.91 (0.11)	1.65 (0.13)	1.95 (0.06)	T vs. D, *p* = 0.163 T vs. L, *p* = 0.762 **D vs. L,** ***p*** = **0.047**

Models were run in three separate substantive peer outcome blocks (i.e., peer characteristics, peer status, and peer relationship quality) while controlling for demographics (i.e., age, gender, race, income, and site) and early adversities. Bold indicates significant effects

*D* deprivation ACE class, *L* low ACE class, *T* threat ACE class

#### Peer characteristics

The threat class reported significantly adverse outcomes regarding peer characteristics than the low ACE class. Specifically, youth in the threat class reported higher peer substance use (1.89 vs. 1.48, *p* = 0.011) and peer delinquency (2.53 vs. 1.86, *p* = 0.002) alongside lower positive peer behaviors (4.28 vs. 4.51, *p* = 0.059) than the low ACE class. No significant differences were found between the deprivation and low ACE classes or between the threat and deprivation classes for peer characteristics.

#### Peer status

The threat class showed lower peer status compared to the low ACE class. Specifically, youth in the threat class were described by their teachers as having lower peer popularity (1.97 vs. 2.31, *p* = 0.001) and higher peer aggression (1.39 vs. 1.07, *p* = 0.041) compared to youth in the low ACE class. Further, youth in the threat class self-reported higher peer victimization (0.69 vs. 0.24, *p* = 0.001) than youth in the low ACE class. No significant differences were found between the deprivation and low ACE classes or between the threat and deprivation classes regarding peer status.

#### Peer relationship quality

The deprivation class reported lower mean levels on all peer relationship quality variables than the low ACE class and on three variables than the threat class. Specifically, youth in the deprivation class reported lower peer companionship (1.62 vs. 1.99, *p* = 0.004), conflict (0.85 vs. 1.13, *p* = 0.009), satisfaction (2.63 vs. 2.93, *p* = 0.015) and intimacy (1.65 vs. 1.95, *p* = 0.047) than the low ACE class. Further, compared to the threat class, the deprivation class reported lower peer companionship (1.62 vs. 1.92, *p* = 0.063), conflict (0.85 vs. 1.24, *p* = 0.005), and satisfaction (2.63 vs. 2.97, *p* = 0.021). No significant differences were found between the threat and low ACE classes for peer relationship quality.

## Discussion

The associations between adolescent ACEs and peer relationships are critical yet complex and further obscured by several limitations in the literature, which include (1) investigating ACEs separately, (2) lacking examination of ACEs in adolescence, (3) predominantly cross-sectional designs, and (4) lacking attention to the multifaceted nature of peer relationships. The current study addressed these shortcomings by examining the co-occurring ACE patterns at age 12 and their associations with peer relationship outcomes at age 16 while controlling for demographics and early adversities. Three distinct ACE classes (i.e., threat, deprivation, and low ACEs) with unique implications for peer relationships were identified. Youth in the threat class were more likely to have adverse outcomes in terms of peer characteristics and status. In contrast, youth in the deprivation class tended to report affected peer relationship quality. These findings advance knowledge on the complex associations between adolescent ACEs and peer relationships and support the growing calls to study ACEs during adolescence and consider their co-occurrence (e.g., Hawes & Allen, 2023).

### Early Adolescent ACE Classes and Demographic Characteristics

The ACEs experienced in early adolescence (age 12) clustered into three distinct classes: threat, deprivation, and low ACEs. This number of ACE classes is on the lower end of the range reported in the literature (i.e., 2–8 classes, with most studies reporting 3–5 classes) but is consistent with fewer ACE classes being identified when research focuses on ACEs in a specific developmental period (vs. measuring lifetime ACEs; Wang et al., 2024b). The nature of the ACE classes found in this study overlaps with yet differs from the ACE classes reported in previous LCA studies, which aligns with the notion that ACE classes identified via LCA are often study-specific (Baldwin et al., 2023). The current study advances the ACE literature using LCA because it utilized 10 established ACE indicators, as defined in the CDC-Kaiser ACE study, to model ACE classes, which enhances the comparability of the ACE classes found in this study to classes reported in other research.

LCA has gained popularity in the ACE literature because of its ability to capture ACEs’ co-occurring nature. However, one of its limitations lies in the subjectivity in labeling and interpreting classes (Wang et al., 2024b). This study used theory (i.e., the DMAP model; McLaughlin et al., 2014) to inform the labeling of ACE classes, which offers some guidance for addressing LCA’s limitations while leveraging its strengths in studying ACEs. The identification of threat and deprivation classes also adds to the growing evidence supporting the DMAP’s applicability in behavioral science (McLaughlin, 2016), particularly concerning ACEs in early adolescence. These findings further underscore the need to move beyond the traditional specificity and cumulative models in ACE research, given the theoretical and methodological evidence supporting the significance of capturing distinct ACE classes.

The findings on the demographic characteristics of the ACE classes at age 12 align with and extend the literature. Specifically, this study found youth from higher-income families were less likely to be in the deprivation class (than the low ACE class) and less likely to be in the threat class (than the deprivation class). This aligns with the literature that suggests higher income is a protective factor against profiles of high ACEs (Liu et al., 2018; Wolff et al., 2018). Further, females were less likely to be in the threat class, which might be explained by females being less likely to report witnessing or experiencing violence (Parnes & Schwartz, 2022), although their overall ACE profiles tend to be more complex (Haahr-Pedersen et al., 2020).

Black youth were less likely to be in the threat or deprivation classes than the low ACE class, as compared to their White counterparts. This finding supports the recent literature that shows racially minoritized youth experience different ACE configurations than their White counterparts and are less likely to be in classes with elevated ACEs (e.g., Wolff et al., 2018). However, this finding seems to contradict the traditional cumulative ACE model, which suggests that racially minoritized youth experience higher rates of ACEs (e.g., Felitti et al., 1998). Further, no significant differences between Latinx and White youth were found in relation to the ACE classes in this study, which contradicts previous research (e.g., Maguire-Jack et al., 2020; Wolff et al., 2018). While such discrepancies could be caused by sample differences (e.g., this study analyzed a high-risk sample), it highlights the need to examine the demographic characteristics of the identified ACE classes instead of generalizing the literature on ACE prevalence rates and demographic variables. Notably, the demographic variables examined in this study were at the individual or household levels. Additional factors beyond the household, such as safe neighborhoods and racial discrimination, can affect youth’s risks for ACEs or complicate ACEs’ implications for youth development (i.e., the social determinants of health framework; McEwen & Gregerson, 2019).

### Associations Between Early Adolescent ACE Classes and Later Peer Relationships

Peers form an essential “community” to facilitate trauma recovery for adolescents (SAMHSA, 2014; Wang et al., 2024a). Therefore, it is critical to understand peer relationships among youth with trauma (or ACEs). The findings suggest the threat and deprivation classes have unique implications for peer relationships. Youth in the threat class tended to have adverse outcomes in terms of peer characteristics and status. In contrast, youth in the deprivation class were more likely to report affected peer relationship quality. This finding extends the prior literature that investigated ACEs primarily via the specificity model and suggested ACEs were negatively related to peer status, but their associations with peer characteristics and peer relationship quality were inconclusive (Wang et al., 2024a).

Specifically, this study found early adolescents in the threat class, but not their counterparts in the deprivation class, were more likely to be friends with peers having negative characteristics, namely peers who had higher levels of substance use and delinquency but lower levels of positive behaviors. This finding advances the prior literature that the associations between ACEs and peer characteristics were inconclusive (e.g., 17 studies reported negative links and 12 suggested nonsignificant associations; Wang et al., 2024a). A notable distinction between this study and the prior literature is that the current study examined the implications of co-occurring ACE patterns, whereas the prior literature primarily assessed ACEs through the specificity model. Such discrepancies emphasize the need to consider the co-occurring patterns of ACEs when examining their implications for peer characteristics. Understanding the associations between ACE classes and peer characteristics is critical because having friends who engage in negative behaviors increases the likelihood that youth will engage in similar behaviors (i.e., the socialization effect; Henneberger et al., 2021), which can further negatively impact their development. In contrast, having friends involved in positive behaviors can bolster youth resilience and facilitate positive development (Walters, 2020).

Regarding peer status, this study found that the negative association between ACEs and peer status, as documented in the prior literature (Wang et al., 2024a), only applied to youth in the threat class. Specifically, youth in the threat class tended to have a greater likelihood of being perceived as less likable, acting aggressively toward peers, and being victimized by peers. In contrast, youth in the deprivation class did not differ significantly from their low-ACE counterparts across all three indicators of peer status. This finding further underscores the salience of capturing the distinct co-occurring patterns of ACEs in examining their implications for peer relationships. In addition, the finding that youth in the threat class were more likely to be reported as acting aggressively toward and being victimized by peers is particularly concerning because it mirrors the profiles of bully-victims—a group at heightened risks for negative outcomes compared to other individuals involved in bullying (Kennedy, 2021). Further, this finding is consistent with recent calls to study the relations between ACEs and bully-victims in adolescence, particularly recent ACEs, because of the potential heterogeneity in how different ACEs relate to bullying involvement (Merrin et al., 2024) and the close links between recent ACEs and bully-victims (Kennedy, 2021). For instance, research shows that bully-victims who have high levels of both bullying perpetration and victimization are twice as likely to have experienced ACEs in the past year compared to individuals with moderate levels of bullying or victimization (Kennedy, 2021).

In terms of peer relationship quality, the findings show an opposite pattern. Youth in the deprivation class, but not their counterparts in the threat class, reported affected peer relationship quality, namely lower levels of peer companionship, satisfaction, intimacy, and conflicts. This finding extends the prior literature that the associations between ACEs and peer relationship quality were inconclusive (e.g., 16 studies reported negative associations and 13 suggested nonsignificant links; Wang et al., 2024a) and reinforces the significance of considering ACEs’ co-occurring nature. Interestingly, youth in the deprivation class reported less peer conflict, which contrasts with previous research using the specificity model, where ACEs were either associated with increased peer conflict (e.g., Narayan et al., 2014) or unrelated to peer conflict (e.g., Yoon et al., 2024). Youth in the deprivation class might be more isolated and withdrawn from peers (Hildyard & Wolfe, 2002), potentially resulting in fewer overall peer interactions, which may explain the lower levels of peer conflict observed in this study.

### Implications of the Findings

This study advances the literature on ACEs and peer relationships methodologically and conceptually. It used a person-centered approach that allowed for the modeling of co-occurring ACEs, whereas prior literature predominantly used the specificity model that assessed ACEs individually. This study also focused on ACEs experienced in early adolescence rather than assessing ACEs that occur anytime throughout childhood (i.e., 0–18 years) or solely in early childhood (i.e., 0–5 years). The findings have several implications for research and practice. First, they add empirical evidence to the recent calls for greater attention to ACEs experienced in adolescence. Early adolescence is a critical developmental period characterized by heightened neuroplasticity, making it a pivotal time to study the impact of ACEs on youth development (Dahl et al., 2018; Hawes & Allen, 2023). Second, the unique implications of threat and deprivation classes for later peer relationships highlight the need for peer relationship researchers to move beyond the traditional specificity or cumulative models when examining the complex associations between ACEs and peer relationships (Wang et al., 2024a). To this end, the person-centered approaches, dimensional models, or a combination of these approaches, like this study or Sisitsky et al. (2023), are viable alternatives. Specifically, this study modeled ACE classes based on specific ACEs but referenced DMAP (i.e., a dimensional model) in naming the ACE classes, and Sisitsky et al. (2023) modeled ACE profiles based on ACE dimensions.

The findings also have practical implications. The finding that youth developmental outcomes, such as peer relationships, are differentially related to clusters of ACEs suggests the need to screen and discover ACEs that may co-occur to gain a holistic understanding of youth’s ACE profiles. For instance, for youth who are referred for intervention due to emotional neglect, it would be critical to know if they have also experienced other ACEs and what the other ACEs are (e.g., sexual abuse and physical neglect). This more holistic understanding of youth’s ACE profiles may help practitioners reach a more accurate conceptualization of youth’s potential functioning. Otherwise, practitioners might have an incomplete understanding of youth functioning impairment related to ACEs. In this same example of youth reporting emotional neglect, practitioners might think of them as having lower peer status (Wang et al., 2024a). However, the youth’s peer status may not be affected because they have a deprivation ACE profile (i.e., elevated levels of emotional and physical neglect along with low levels of physical, sexual, and emotional abuse). Of note, practitioners must create a safe environment that encourages disclosure and prevents re-traumatization in order to accurately and comprehensively identify youth ACE profiles. Practitioners must also respond appropriately to the possible emotional reactions youth may experience when sharing their ACEs. Simply gathering information without offering support can re-traumatize youth and exacerbate ACEs’ adverse impact on youth development.

The finding that ACE clusters relate to peer relationships differentially also underscores the need to tailor trauma-informed care (vs. a one-size-fits-all approach) because youth developmental outcomes might be differentially affected by various ACE clusters and thus have differential needs for interventions and support. Trauma-informed care includes programs, organizations, or systems that realize the impact of trauma, recognize the symptoms of trauma, respond by integrating knowledge about trauma into policies and practices, and seek to resist re-traumatization (SAMHSA, 2014). There is considerable variability among trauma-informed interventions in schools, and little is known about what the essential components are and how they may work differently for various students (Avery et al., 2021). In the case of fostering healthy peer relationship development among youth who have experienced ACEs (or trauma), youth in the threat class might benefit more from support to improve their peer status and facilitate affiliations with peers engaging in positive behaviors. In contrast, youth in the deprivation class would benefit from resources to improve their peer relationship quality. Notably, the well-being of youth with ACEs is subjected to a host of environmental factors beyond the ACEs examined in this study, such as safe neighborhoods and racial discrimination (McEwen & Gregerson, 2019). Tailored trauma-informed care needs to consider such factors to be effective.

### Limitations and Future Directions

The findings should be considered along with limitations and future directions. First, the study sample is somewhat homogeneous, as LONSCAN participants were children at high risks for, or having a history of, maltreatment. This homogeneity might explain the limited variability on some of the ACEs, such as caregivers treated violently, parental separation, and household incarceration. Future research employing population- or community-based samples could allow for more heterogeneity in sample characteristics and might facilitate the identification of additional ACE classes, such as unpredictability (McLaughlin et al., 2021). However, this high-risk sample provided a unique opportunity to examine the complex co-occurring ACE patterns around age 12, which may be challenging to capture in normative samples due to lower response rates (Wolff et al., 2018).

Second, this study advances previous cross-sectional research on ACEs and peer relationships by relating ACE classes at age 12 to peer relationship outcomes at age 16. However, the selection of these two specific ages was partially driven by data availability in LONSCAN (i.e., age 12 is the only early adolescence time point with available data on all 10 ACEs, and age 16 is the only time point after age 12 with available data on all peer relationship variables of interest) and is limited to two waves of data. Future longitudinal research on ACE classes at early adolescence time points other than age 12, as well as peer relationships in middle adolescence other than age 16 or late adolescence, would enrich the literature. In addition, longitudinal studies of three or more waves are especially needed.

Third, informed by the social learning theory, this study leveraged the two points of the LONSCAN data in adolescence to examine the implications of early adolescent ACEs for later peer relationships. However, youth’s peer relationships might further affect their risks of experiencing ACEs. For instance, Meinck et al. (2017) found girls who perceived high levels of peer support at baseline were less likely to report sexual abuse at the 1-year follow-up. Future research examining the reciprocal associations between ACEs and peer relationships may meaningfully contribute to the literature.

Fourth, the unique implications of the threat and deprivation ACE classes for peer relationships were primarily based on their comparisons with the low ACE group. Direct comparisons between the threat and deprivation classes were significant for only three of the 10 peer relationship outcomes, likely due to the small sizes of these two subgroups (i.e., threat, *n* = 174; deprivation, *n* = 97). Future studies with larger sample sizes will allow for more robust comparisons between the threat and deprivation classes.

Lastly, this study followed a holistic approach and examined three salient aspects of peer relationships, including both positive and negative indicators, in relation to earlier co-occurring ACE patterns. However, it is unclear how other peer relationship aspects, such as peer relationship quantity, peer support, or peer influence (Wang et al., 2024a), are affected by the co-occurring ACE patterns in early adolescence. Further, it is unclear how peer relationships further affect the well-being of youth experiencing specific ACE clusters in early adolescence (e.g., mediation and moderation). Future research exploring these areas would advance the literature regarding the complex links between adolescent ACEs and peer relationships and provide insight into supporting adolescent trauma recovery by engaging their peers (SAMHSA, 2014).

## Conclusion

Research indicates complex implications of ACEs for adolescent peer relationships, which is further obscured by several methodological limitations (e.g., lacking consideration of ACEs’ co-occurring nature and ACEs experienced in adolescence). Thus, the current study explored early adolescents’ co-occurring ACE patterns and their implications for later peer relationships while controlling for demographics and early adversities. This study identified three distinct ACE classes experienced around age 12 (i.e., threat, deprivation, and low ACEs) that were characterized by gender, race, income, and early adversities. These ACE classes had further unique implications for peer relationships at age 16: (1) the threat class was associated with negative peer characteristics and lower peer status, and (2) the deprivation class was related to differences in peer relationship quality. These findings underscore the need for researchers and practitioners to pay attention to ACEs’ co-occurring nature and suggest the need to tailor trauma-informed care accordingly. Findings also highlight the salience of studying ACEs that occur in early adolescence.

## Supplementary information

Supplemental Materials
