# Supplementary material for: Co-occurring Early Adolescent ACEs and Associations With Later Peer Relationships
Source: J Youth Adolesc. 2025 Feb 13;54(7):1827–44. doi: 10.1007/s10964-025-02157-0 (PMC12246021; doi:10.1007/s10964-025-02157-0)
Supplement: Supplementary file 1 — Supplemental Materials [file 10964_2025_2157_MOESM1_ESM.docx]

Supplemental Materials for:

**Co-occurring Early Adolescent ACEs and Associations With Later Peer Relationships**

In Journal of Youth and Adolescence

Joy Huanhuan Wang^1^, Gabriel J. Merrin^2^, Xiafei Wang^3^, Qingyang Liu^2^, Sarah M. Kiefer^4^

^1^ College of Education, Texas Tech University

^2^ Department of Human Development and Family Science, Syracuse University

^3^ School of Social Work, Syracuse University

^4^ College of Education, University of South Florida

**Author Note**

Correspondence concerning this article should be addressed to Joy Huanhuan Wang, College of Education, Texas Tech University, 3002 18th Street, Lubbock, TX 79409, USA. Email: [joy.wang@ttu.edu](mailto:joy.wang@ttu.edu)

**Appendix A**

*The Specific LONGSCAN Items Used in Assessing ACEs and Peer Relationships*

| ACEs | Corresponding LONSCAN items |
| --- | --- |
| Physical abuse ^a^ | PHYA1, PHYA4, PHYA5, PHYA8, PHYA9, PHYA12, PHYA13, PHYA16, PHYA17, PHYA20, PHYA21, PHYA24, PHYA25, PHYA28, PHYA33, PHYA36, PHYA41, PHYA44, PHYA45, PHYA48, PHYA49, PHYA52, PHYA53, PHYA56, PHYA57, PHYA60, PHYA61, PHYA64, PHYA65, PHYA68, PHYA69, PHYA72 |
| Sexual abuse ^b^ | SARA1, SARA4, SARA5, SARA8, SARA9, SARA12, SARA13, SARA16, SARA17, SARA20, SARA21, SARA24, SARA25, SARA28, SARA29, SARA32, SARA33, SARA36, SARA37, SARA40, SARA41, SARA44, SARA45, SARA48 |
| Emotional abuse ^c^ | PSMA1, PSMA1C, PSMA2, PSMA2C, PSMA3, PSMA3C, PSMA5, PSMA5C, PSMA6, PSMA6C, PSMA7, PSMA7C, PSMA8, PSMA8C, PSMA9, PSMA9C, PSMA10, PSMA10C, PSMA11, PSMA11C, PSMA12, PSMA12C, PSMA13, PSMA13C, PSMA14, PSMA14C, PSMA15, PSMA15C, PSMA17, PSMA17C, PSMA18, PSMA18C, PSMA19, PSMA19C, PSMA20, PSMA20C, PSMA24, PSMA24C, PSMA26, PSMA26C |
| Physical neglect | AMPA6B, AMPA10B, AMPA12B, AMPA19B, AMPA20B, AMPA21B, AMPA22B |
| Emotional neglect | AMPA1B, AMPA2B, AMPA5B, AMPA7B, AMPA15B, AMPA16B, AMPA18B |
| Caregiver treated violently | CTPC4, CTPC5, CTPC9, CTPC11, CTPC14, CTPC17, CTPC19, CTPC22, CTPC23, CTPC27, CTPC31, CTPC37 |
| Family substance misuse | RBFA4, RBFA5, RBFA6, RBFA7, RBFA9 |
| Maternal depressive symptoms | DEPB1, DEPB2, DEPB3, DEPB4, DEPB5, DEPB6, DEPB7, DEPB8, DEPB9, DEPB10, DEPB11, DEPB12, DEPB13, DEPB14, DEPB15, DEPB16, DEPB17, DEPB18, DEPB19, DEPB20 |
| Parental separation | FCHB2C, FCHB2D, FCHB3C, FCHB3D, FCHB4C, FCHB4D, FCHB5C, FCHB5D, FCHB6C, FCHB6D, FCHB7C, FCHB7D, FCHB8C, FCHB8D, FCHB9C, FCHB9D, FCHB10C, FCHB10D, FCHB11C, FCHB11D, FCHB12C, FCHB12D, FCHB13C, FCHB13D, FCHB14C, FCHB14D, FCHB15C, FCHB15D |
| Household incarceration ^d^ | LECC20, LECC20A, LECC20B, LECC20C |
| Peer substance use | RBFA15, RBFA16, RBFA19, RBFA20, RBFA21, RBFA22 |
| Peer delinquency | RBFA17, RBFA18, RBFA23, RBFA24, RBFA25, RBFA26, RBFA27 |
| Positive peer behavior | RBFA10, RBFA11, RBFA12, RBFA13, RBFA14 |
| Peer popularity | TRPC1, TRPC2, TRPC6, TRPC7 |
| Peer aggression | TRPC4, TRPC5 |
| Peer victimization ^e^ | YPVA3; YPVA4A, YPVA4B, YPVA4C, YPVA4D, YPVA4E, YPVA4F, YPVA4G, YPVA4H, YPVA4I, YPVA4J, YPVA4K; YPVA5A3, YPVA5A4, YPVA5B3, YPVA5B4, YPVA5C3, YPVA5C4, YPVA5D3, YPVA5D4, YPVA5E3, YPVA5E4, YPVA5F3, YPVA5F4, YPVA5G3, YPVA5G4, YPVA5H3, YPVA5H4, YPVA5I3, YPVA5I4, YPVA5J3, YPVA5J4, YPVA5K3, YPVA5K4. |
| Companionship | NRIA4C, NRIA4D, NRIA8C, NRIA8D, NRIA12C, NRIA12D |
| Conflict | NRIA5C, NRIA5D, NRIA9C, NRIA9D, NRIA13C, NRIA13D |
| Satisfaction | NRIA6C, NRIA6D, NRIA11C, NRIA11D, NRIA14C, NRIA14D |
| Intimacy | NRIA7C, NRIA7D, NRIA10C, NRIA10D, NRIA15C, NRIA15D |

*Note.* ACEs = adverse childhood experiences; LONSCAN = Longitudinal Studies of Child Abuse and Neglect.

^a^ PHYA1 branches for PHYA4, PHYA5 for PHYA8, and so on.

^b^ SARA1 branches for SARA4, SARA5 for SARA8, and so on.

^c^ PSMA1 branches for PSMA1C, PSMA2 for PSMA2C, and so on.

^d^ LECC20 branches for LECC20A, LECC20B, LECC20C.

^e^ YPVA3 branches for YPVA4A through YPVA4K, and YPVA4A through YPVA4K branch for YPVA5A1 through YPVA4K.

**Table S1**

*Comparisons Between the Baseline and Analytic Samples*

| Variable | Baseline sample | Analytic sample |
| --- | --- | --- |
|  | *N* = 1,354 | *N* = 883 |
| Race |  |  |
| Black | 721 (53.2%) | 487 (55.2%) |
| Hispanic | 97 (7.2%) | 56 (6.3%) |
| Multiracial | 161 (11.9%) | 100 (11.3%) |
| Other | 20 (1.5%) | 11 (1.2%) |
| White | 354 (26.1%) | 229 (25.9%) |
| Gender |  |  |
| Female | 697 (51.5%) | 444 (50.3%) |
| Male | 657 (48.5%) | 439 (49.7%) |
| Site |  |  |
| East | 282 (20.8%) | 185 (21.0%) |
| Midwest | 245 (18.1%) | 132 (14.9%) |
| Northwest | 254 (18.8%) | 176 (19.9%) |
| South | 243 (17.9%) | 164 (18.6%) |
| Southwest | 330 (24.4%) | 226 (25.6%) |

**Figure S1**

*Plotted Endorsement Probabilities for Each ACE by Class*

Physical Abuse

Sexual Abuse

Emotional Abuse

Physical Neglect

Emotional Neglect

Caregiver Treated Violently

Family Substance Misuse

Maternal Depressive Symptoms

Parental Separation

Household Incarceration

**Figure S2**

*Plotted Means by Peer Relationship Outcome Blocks*

Peer Characteristics

Peer Status

Peer Relationship Quality
